# A multi-stage dataset for banana bunch detection and harvesting decision support

**DOI:** 10.1016/j.dib.2025.112337

**Published:** 2025-12-03

**Authors:** Preety Baglat, Fábio Mendonça, Sheikh Shanawaz Mostafa, Fernando Morgado-Dias

**Affiliations:** a University of Madeira, 9000-082 Funchal, Portugal; b Interactive Technologies Institute (ITI/LARSyS and ARDITI), 9020-105 Funchal, Portugal

**Keywords:** Datasets, Machine learning, Computer vision, Images, Agriculture, Banana

## Abstract

This study introduces a multi-part dataset designed to support the development of artificial intelligence models for banana bunch detection and harvesting decision-making. The dataset includes images collected from four different fields in Madeira Island, Portugal, under varying environmental conditions. It is divided into three core subsets, namely, a detection dataset annotated using You Only Look Once (YOLO) format (2179 images labelled for bunch and flower bud detection), a harvesting classification dataset labelled by expert teams as “Cut”“ and “Keep” (2685 images, with 1143 labelled as “Cut” and 1542 as “Keep”) and an expert opinion dataset where images were classified by human experts into three decision categories: “Cut now”, “Keep for next cut” and “Wait more than three weeks” (400 images, with 100 samples evaluated by each of four expert cutters, capturing all three decision categories) These datasets enable the creation and benchmarking of computer vision models and allow for expert consensus analysis.

Specifications Table

**Table:** 

Subject	Computer Sciences
Specific subject area	Machine learning, deep learning, computer vision, bunch detection and banana bunch harvesting, agricultural engineering
Type of data	Images JPEG (Joint Photographic Experts Group); Text files (YOLO-format annotations in .txt); Comma Separated Values (CSV) file (converted bounding box annotations for detection dataset).
Data collection	This dataset was collected over 29 visits to banana plantations in Madeira, Portugal, during real harvesting conditions. High-resolution Red, Green, and Blue (RGB) images were taken manually from mobile phones, similar to how the harvesting experts observe them on the field. All the data in this dataset comes only from Cavendish cultivar subgroup of Musa acuminata, which are the main type of bananas grown and harvested in this region.
Data source location	Institution: GESBA (Madeira Banana Sector Management Company), Madeira, Portugal. Sampling region: Madeira Island, Portugal Collected from four different fields: Lugar de Baixo (32.679952^◦^ N 17.086920^◦^ W); Ponta do Sol (32.680026^◦^ N and 17.086764^◦^ W); Santo António (32.667100^◦^ N, −16.955559^◦^ W) and Câmara de Lobos (32.656786^◦^ N, −16.964987^◦^ W)
Intended use (Image task)	D1: Detect bunch in full image D2: Predict Cut or Keep on cropped bunch D3: Expert agreement and decision-cue analysis
Data accessibility	Data accessibility: Mendeley Data Repository and Zenodo Direct URL to all datasets: https://github.com/Preety36/banana-harvesting-datasets Data Links and DOIs: Dataset (D1): Banana Bunch Detection Dataset (Zenodo) https://doi.org/10.5281/zenodo.15642838 Dataset (D2): Banana Bunch Harvesting Dataset (Mendeley) https://doi.org/10.17632/kjrsb7ztr9.1 Dataset (D3): Banana Bunch Harvesting Expert Dataset (Mendeley) https://doi.org/10.17632/kk88rgfr55.1
Related research article	P. Baglat, A. Hayat, S. S. Mostafa, F. Mendonça, and F. Morgado-Dias, “Comparative analysis and evaluation of YOLO generations for banana bunch detection,” Smart Agricultural Technology, vol. 12, Dec. 2025, doi:10.1016/j.atech.2025.101100. A. Hayat, P. Baglat, F. Mendonça, S. S. Mostafa, and F. Morgado-Dias, “Machine learning system for commercial banana harvesting,” Engineering Research Express, vol. 6, no. 3, Sep. 2024, doi:10.1088/2631-8695/ad5cd2.

## 1 Value of the Data

This dataset was gathered from real-world banana plantations in Madeira, Portugal**,** through 29 visits under varying lighting and environmental conditions, very representative and reliable for practical uses. It includes three datasets:

1. **D1 (Detection):** 2179 high-quality images and labels of banana bunches.
2. **D2 (Classification):** 2685 cropped images categorized with 1143 labeled as “Cut” and 1542 as “Keep” supporting harvest readiness classification.
3. **D3 (Expert Opinion):** 400 images assessed by 4 specialist harvesters with specific labels such as “cut now”, “keep next”, and “wait 3 weeks”.

The data was cleaned and processed manually**,** a process that involved the elimination of sensitive details (e.g., label plates) and unclear images (e.g., images with too many obstacles or out of focus). This dataset allows researchers to create and assess detection and classification models for banana harvesting, measure human-artificial intelligence agreement, and enhance decision-support tools for intelligent agriculture. To the best of our knowledge, none of the datasets currently published are specially designed for harvesting readiness.

## 2 Background

Bananas are among the most cultivated and consumed fruits globally, playing an important role in food security and local economies in many tropical and subtropical regions [1]. Despite its importance, banana harvesting still relies heavily on manual labor and expert judgment, which can be subjective, inconsistent, and resource-intensive [2,3]. Recent advancements in artificial intelligence and computer vision have opened new possibilities for automating parts of the harvesting process, particularly through object detection and maturity classification models. However, the progress of such systems is delayed by the limited availability of high-quality, publicly accessible datasets that reflect real-world conditions [[4], [5], [6], [7]].

While many existing agricultural datasets focus on disease recognition or yield estimation, there is a gap in datasets that support harvesting decision-making [6]. In the context of bananas, most prior work has targeted the detection of bunches, buds, or stalks using variants of the YOLO architecture, often under ideal lighting or experimental settings [8,9]. Moreover, many of these studies used datasets that are either small, synthetic, or unavailable for public use, restricting the reproducibility and comparability of their models. Recent evaluations report YOLO-based detection performance for D1 and deep-classifier performance for D2**,** providing baseline expectations in this domain [3,10]. In addition, recent banana-specific resources include a bunch-level image-and-video dataset that supports variety classification and grading from in-orchard capture [11], and a banana dataset focused on carbide vs. non-carbide and ripening stage analysis for quality and post-harvest applications [12]. Compared with these resources, the D1 dataset has larger samples of banana bunches, and D2 is unique as banana bunches are expert labeled to directly support harvest decision.

Existing studies include Transformer-based detectors (e.g., DETR) [13] and Vision Transformers (ViT) for classification [14], updated ConvNet families (e.g., ConvNeXt) [15], and self-supervised pretraining approaches (e.g., contrastive or masked-image methods) that improve robustness when data vary in lighting, viewpoint, and background [[16], [17], [18]]. Accordingly, D1 supports modern detectors, D2 supports classifier baselines (including ViT), and D3 provides feedback on the quality of expert labeling. The datasets here (D1, D2 and D3) are compatible with these families and support both supervised and self-supervised pipelines.

To address this challenge, a new dataset has been created from banana plantations in Madeira Island, Portugal, covering diverse real-world field conditions. It supports multiple tasks, including object detection, harvesting readiness classification, and consensus modeling. Along with the dataset, details on acquisition, annotation methodology, and expert-grounded labeling are provided. The dataset is openly available on GitHub [19], encouraging further research and benchmarking for precision agriculture applications.

## 3 Data Description

To provide a comprehensive understanding of the data used in this study, this section introduces three datasets. Each one was created with a different purpose, whether it is detecting banana bunches, deciding if they are ready to harvest, or capturing expert opinions. Together, they offer a wide range of images and labeling styles that are useful for building and testing AI tools for banana harvesting. These datasets are based entirely on Cavendish cultivar subgroup of Musa acuminata bananas, which are the most commonly grown variety in the region.

All three subsets use Cavendish bananas from the same fields and smartphones: Samsung Galaxy A12, Galaxy Note 9, OnePlus 9, and iPhone 12, to match real deployment devices used by farmers and harvesters. Banana harvesting is done by farmers and harvesters who carry smartphones, not professional cameras. Using phones to collect images matches real world use (what users will operate), keeps the process safe and practical in the field, and is affordable and easy to replicate. If images were captured with professional cameras, the trained models could fail to generalize when used on smartphone photos later. Training in phone images avoids this gap and improves real-world performance. Data collected over 29 field visits at different times of day with varied weather conditions, using hand-held smartphone cameras at typical distances of 1–3 m and varied viewing angles, stored as JPEG. D1 (Detection) was used to detect the banana bunch and identify the bunch in focus. To maintain quality images, 662 low-visibility images were identified and removed manually, retaining 2179 high quality annotated images. In D2 (classification) was used for harvesting classification, where number plates were used in the field to track samples (e.g., X001 for Cut, A001 for Keep); plates were later removed during image pre-processing, so models learn from only fruit cues. In a few images, a tiny gray piece of the metal clip may remain at the bottom border of images. The expert generally makes decisions about the harvesting readiness based on the upper section of banana bunch, and edges of the banana fingers, hence those pins do not add significant noise to the image. Also, the harvesting classification decision is always made on green banana bunches and therefore, impact of color-based noise here is minimal. Cropping further to remove them would often cut away important parts of the bunch (especially the top, where key cues like finger fullness, curvature, and color uniformity appear), so conservative crops are kept. Users who want stricter cleaning can apply a thin border mask or simple augmentations (random erasing, occlusion)**.** D3 (Expert opinion) labels the same cropped images into three categories (“Cut now”, “Keep for next cut”, “Wait > 3 weeks”) to measure expert agreement and analyze decision cues**.**

### 3.1 Detection dataset (D1)

**Fig. 1:**
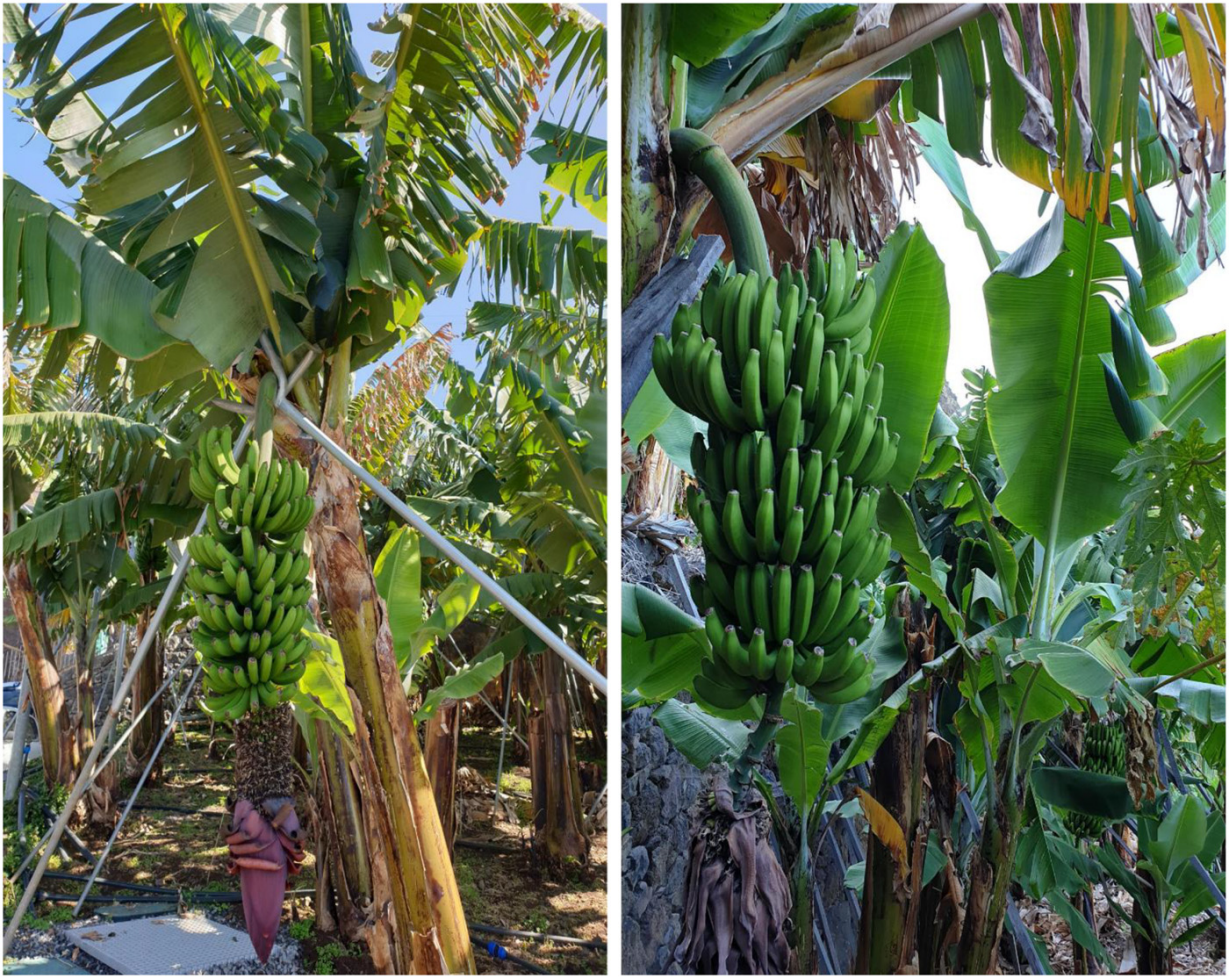
Example images of banana bunches used for detection.

This dataset contains a total of 2841 raw images of banana bunches captured during the year 2022. The photographs were taken in real banana plantations across four different locations on Madeira Island, Portugal, namely, Lugar de Baixo **(**32.679952°- N, −17.086920° W)**,** Ponta do Sol **(**32.680026° N and −17.086764° W)**,** Santo António **(**32.667100° N, −16.955559° W), and Câmara de Lobos **(**32.656786° N, −16.964987° W). Multiple smartphones were used to allow for a range of image qualities and conditions. The main purpose of this dataset is to support object detection tasks, particularly for identifying banana bunches in natural environments. Each image was annotated using standard image labeling tools following the YOLO format**,** which includes drawing bounding boxes around the banana bunches along with their flower buds. During data preparation**,** 662 images were discarded due to poor visibility, caused by factors such as plastic coverings, leaf occlusion, and excessive light exposure. As a result, the final dataset contains 2179 high-quality, annotated images collected during the year of 2022**.** The diversity in lighting, weather conditions, and background clutter in this dataset makes it useful for training and evaluating computer vision models for fruit detection. Examples of banana bunch images are shown in Fig. 1.

### 3.2 Harvesting classification dataset (D2)

This dataset was designed to support classification tasks**,** where the goal is to predict whether a banana bunch is ready to be harvested or not. The data was collected during 29 field visits with banana harvesting teams. The labeling was done in collaboration with field experts. Experts make the decision using simple field cues: edges and shape of the fingers, the condition of the top of the bunch, firmness, color, finger length and overall feel, signs of disease (cut earlier if disease is likely), subtle maturity color within the green range, season and weather conditions**.**

**Fig. 2:**
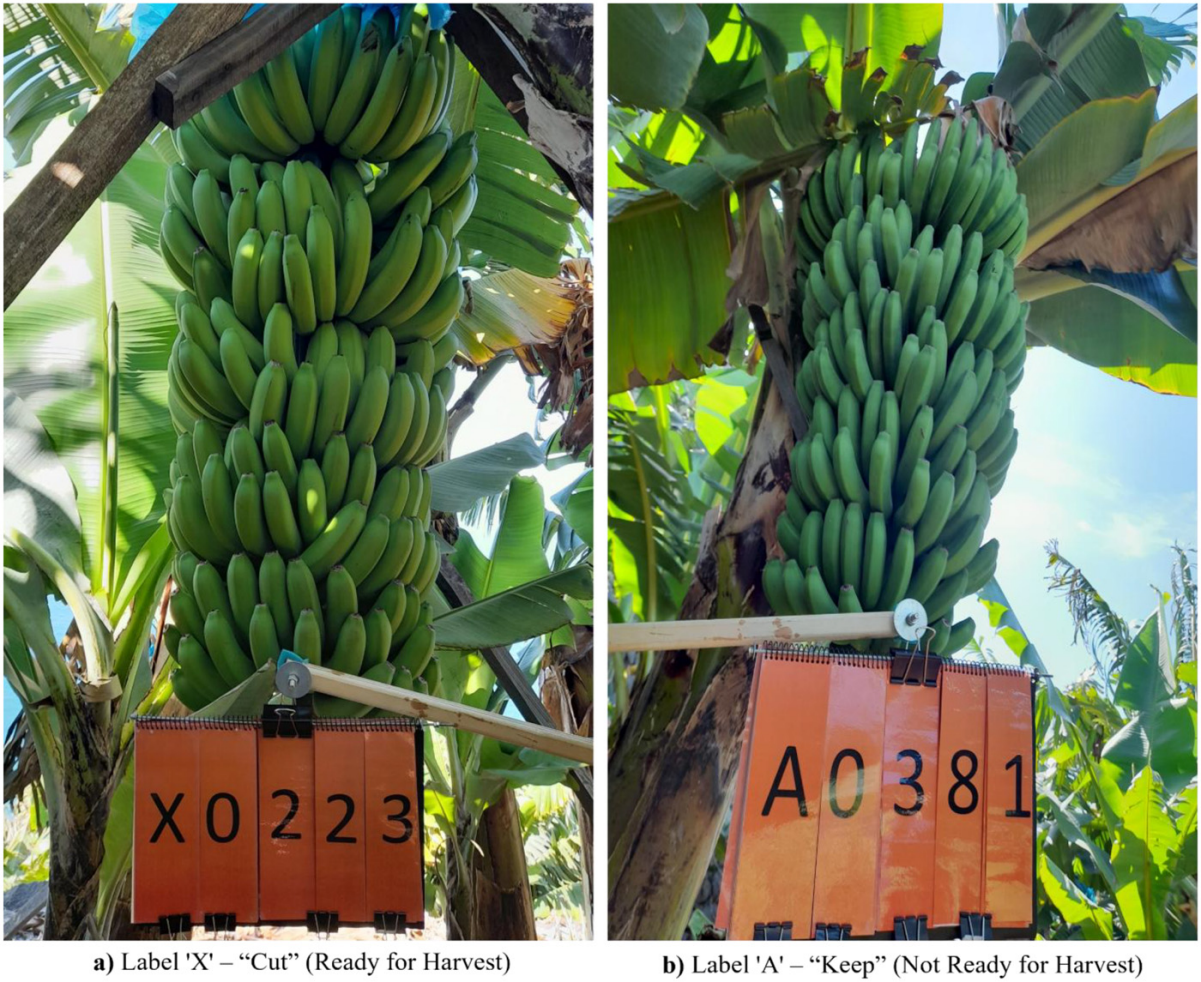
Sample Images of Banana Bunches Labeled as "Cut" (Ready for Harvest) and "Keep" (Not Ready for Harvest).

Each image in this dataset is categorized as either “Cut”, representing bunches that were ready for harvesting at that moment (1143 images), and “Keep”, representing bunches that were not yet ready for harvesting (1542 images). To avoid mixing categories, a two-pass protocol and a number-plate system were used. In each field visit, experts first identified bunches to be harvested (Cut), tagged them *X* + 4-digit ID (e.g., X0223), photographed them, and then harvested them. After finishing the harvesting for the whole field, only the remaining bunches were photographed and tagged *A* + 4-digit ID (e.g., A0386) as Keep. This procedure, together with the “X” and “A” IDs, ensures the same bunch cannot appear in both categories. All photos were captured in RGB and saved in JPEG format. Example images showing this labeling are presented in Fig. 2 in the previous section.

In total, there are 2685 labeled images**.** The fields used for data collection vary in altitude (ranging from 21 to 308 m above sea level) and area (between 21,000 and 308,000 square meters)**.** This variation introduces environmental richness and allows the dataset to represent real harvesting scenarios with seasonal and spatial diversity.

**Fig. 3:**
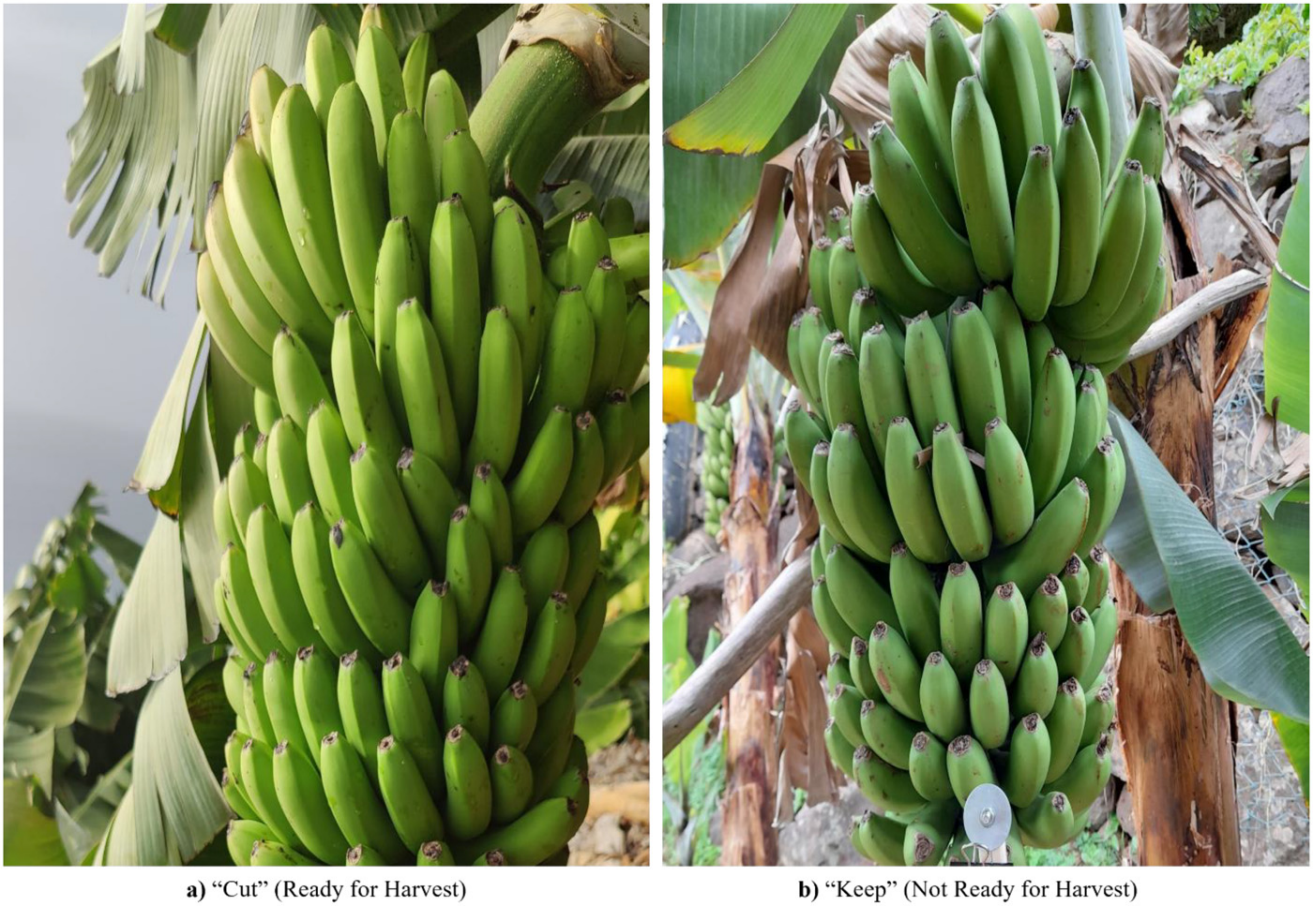
Cropped images of banana bunches used in the final dataset.

To make the dataset suitable for training, an automatic cropping process was applied to remove the number plates and focus only on the banana bunches. This helped prevent the model from learning to classify based on the label text instead of the visual features of the fruit. The system first detected the number plate based on color, then used text recognition based on Easy Optical Character Recognition (EasyOCR) with Character Region Awareness for Text (CRAFT) and Convolutional Recurrent Neural Network (CRNN), to identify the label and finally cropped the image to retain only the region containing the banana bunch. As a result, the final dataset includes clean, focused images of banana bunches without number plates, making it more suitable for training classification models. This preprocessing method is based on the approach described in Hayat et al. [10]. Fig. 3 displays the cropped images included in the final dataset [20]. D2 predicts “Cut” or “Keep” from D1 crops with number plates removed. Any tiny gray piece at the edge is field hardware (clip or support pin), not a label cue, and appears in both classes.

### 3.3 Expert opinion dataset (D3)

**Fig. 4:**
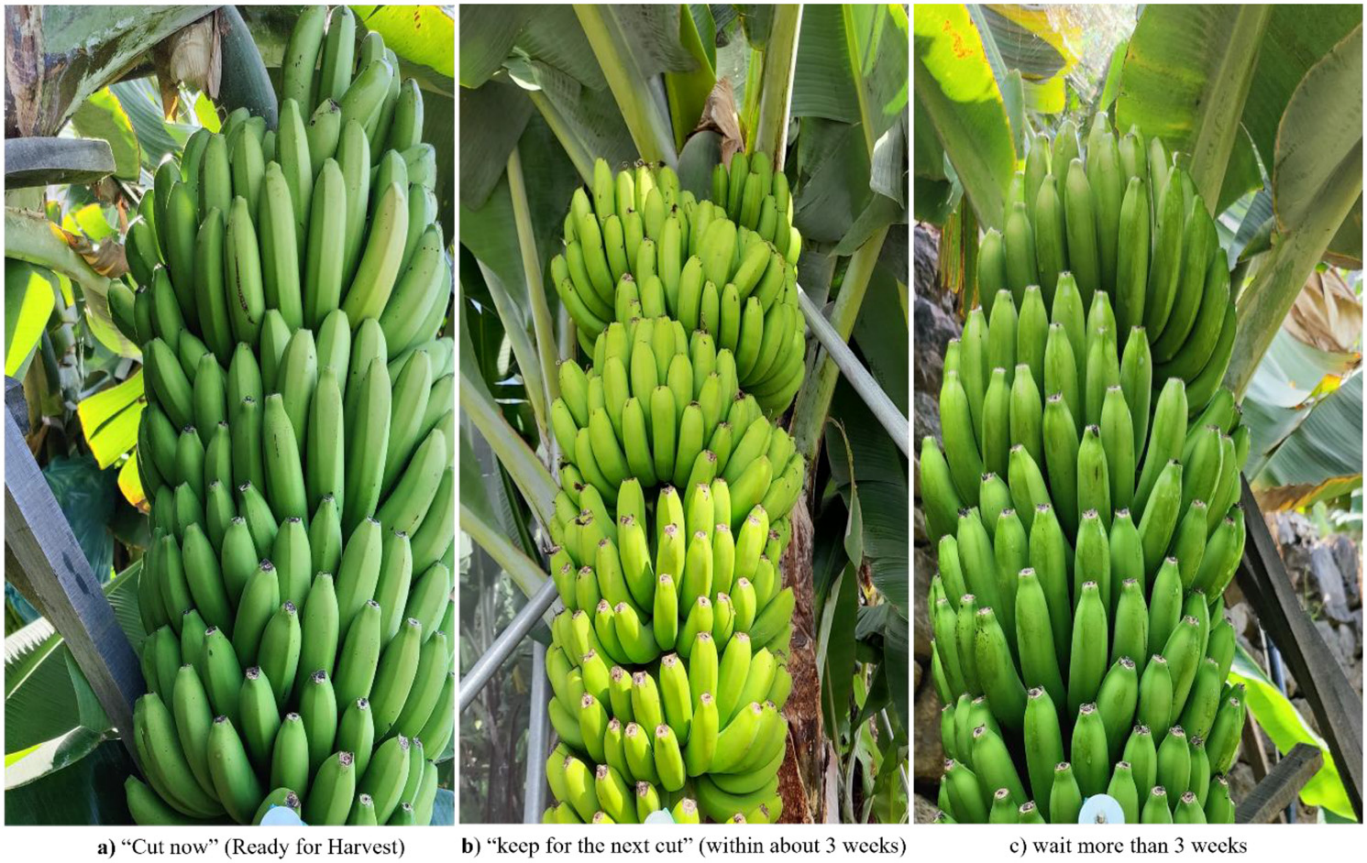
Example images of banana bunches categorized as “Cut now,” “Keep for the next cut,” and “Wait >3 weeks” based on expert harvesting decisions.

To better understand how human experts make harvesting decisions based solely on images, a third dataset was developed. A set of 400 images was randomly selected from the classification dataset and presented to a team of four banana harvesting experts**.** Each expert independently evaluated 100 images using a custom-built, interactive graphical user interface developed with Streamlit [21]**.** Experts were blinded to the original field labels and to each other’s choices, and the image order was randomized for each expert. For each image, the expert had to choose one of the following options: **“**Cut now” if the bunch should be harvested immediately; “keep for the next cut” if the bunch should be harvested in the next field visit (within about 3 weeks); “wait >3 weeks” if the bunch should be kept for a longer time before harvesting. The interface displayed the image on one side of the screen, and the three choices on the other. To ensure consistency in judgment, the experts were told to assume a typical field environment in Madeira with moderate altitude and average sunny weather. D3 is used to quantify expert agreement and analyze decision cues with the three-category labels. For two-class comparisons, a simple mapping can be applied (Cut now to Cut; Keep for next cut/Wait > 3 weeks to Keep); D3 is designed to support consensus or soft-label analyses without reporting numerical agreement rates in this study. Fig. 4 shows sample images representing each of these three categories based on expert decisions. The main details of each dataset, like why it was created, how many images it includes, how the labels were assigned, and where the data was collected, are all shown in Table 1.

**Table 1:** Overview of the Banana Harvesting Datasets and Their Characteristics.

Dataset ID	Name	Purpose	Total Images	Labels	Collection Details	Key Features
**D1**	Detection Dataset	Object detection of banana bunches	2179	YOLO (bunch and flower bud)	2022, Madeira (4 regions), 4 smartphone cameras	High variation in lighting, occlusion, weather; 662 poor-quality images discarded
**D2**	Harvesting Classification	Binary classification (ready/not ready)	2685	“Cut” (1143), “Keep” (1542)	29 field visits; altitude: 21–308 m; area: 21k–308k m²	Seasonally and geographically diverse; field-expert labeled; before/after decision photos
**D3**	Expert Opinion Dataset	Expert consensus on harvest timing	400	“Cut”, “Keep”, “Wait >3 Weeks”	Random sample from D2; 4 experts; labeled via custom Streamlit UI	Multi-label soft decisions; captures subjectivity in field judgment; controlled environment cues

Note: D1 - Detection Dataset, D2 - Harvesting Classification Dataset, D3 - Expert Opinion Dataset.

## 4 Experimental Design, Materials and Methods

**Fig. 5:**
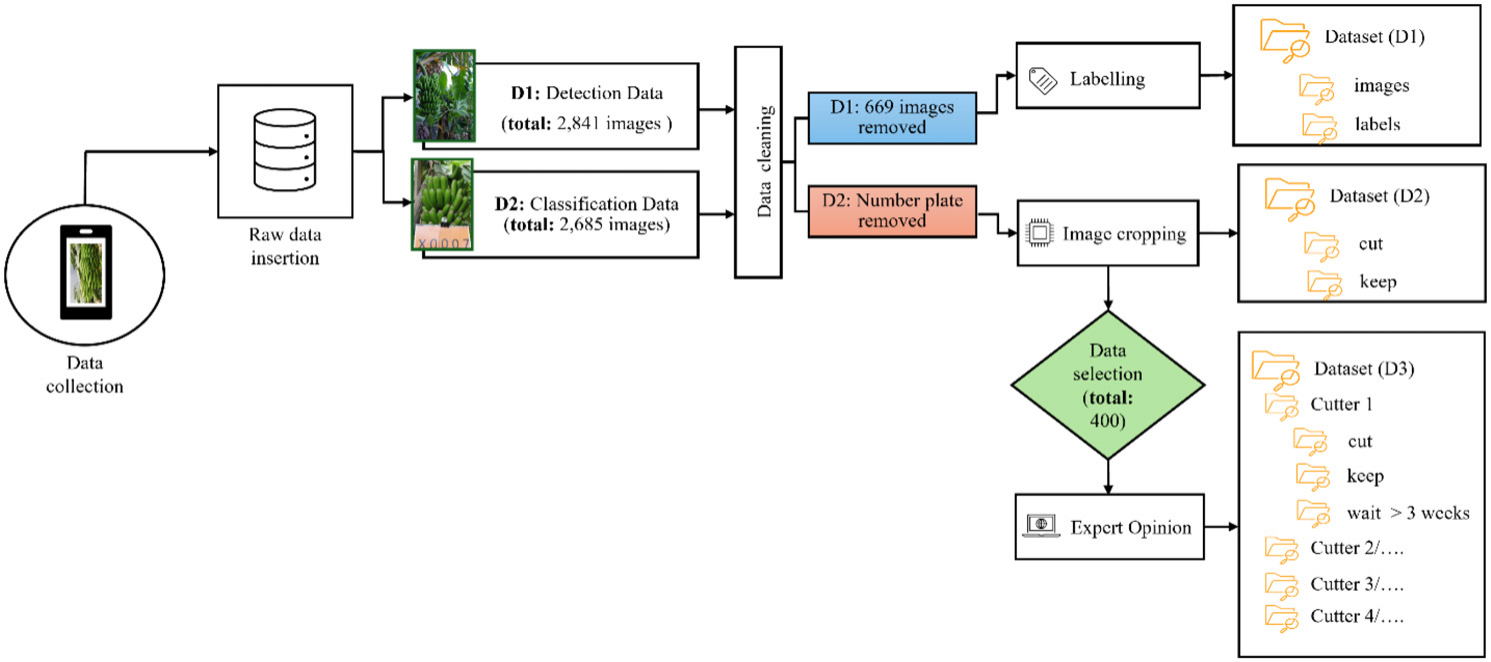
Task-specific dataset structure and processing workflow for banana harvesting.

Data collection was done every two or three weeks to help make sure that all the data is reliable, consistent, and dynamic. All datasets were taken in diverse environmental conditions, such as variable lighting and multiple weather conditions, to make it as broad as possible, helping AI models to train on almost all possible input data. Fig. 5 presents a visual representation of the structure of the dataset.

On the detection subset (D1), YOLOv12n achieves AP^50 test^ 93 % with approximately 5.1 ms per image in edge-like settings [3]. On the harvest-readiness classification subset (D2), DenseNet121 reaches accuracy 83.2 % (Precision 85.4 %**,** recall 82.0 %**,** f1 82.5 % and specificity 83.2 %) using our released split [10]. These results show the datasets work with standard AI models.

To support different machine learning needs, each dataset was stored in an organized folder structure. Only images with visible banana bunches and flower buds were kept, resulting in 2179 detection images for the final dataset. The detection dataset, which includes all high-quality images, was saved in a folder named “Dataset (D1)”, and each image had a corresponding text file in a labels folder. These text files contained manually created bounding boxes in YOLO format, where each label file matches the name of its corresponding image. The bounding boxes were annotated using a dedicated labeling tool [22].

The harvesting classification dataset is stored in a folder named “Dataset (D2)”, which contains two subfolders, specifically, “cut” and “keep”. Each of these folders holds images that represent real harvesting decisions made in the field, “cut” includes images of banana bunches that were harvested on the day of the image capture, while “keep” contained images of bunches that were left on the banana tree for future harvesting date. These images were collected independently during field visits. To prevent any mix-up between the two categories, bunches marked for cutting were photographed first, followed by those that were to be kept, ensuring no overlap. A number plate system was used to visibly tag each bunch during image capture. Cut images were marked with a code starting with the letter ‘X’ and a four-digit number, while keep images were marked with a code starting with ‘A’ and a four-digit number. Some example images using this labeling system are shown earlier in Fig. 2. After collecting, each image was automatically processed to remove any visible number plates used during labeling, so the focus remains entirely on the banana bunch itself. This cleaning step used a combination of color filtering and optical character recognition (using EasyOCR) to detect and crop out the label region, following the approach described by Hayat et al. [10]. The final images were then stored in a ready-to-use structure that allows for direct use in machine learning pipelines. This simple two-folder format is compatible with common tools like PyTorch’s ImageFolder, making it easy for researchers to train machine learning models.

As shown in Fig. 5, D2 is a distinct branch of the dataset pipeline, designed specifically to support classification models that predict whether a banana bunch is ready for harvest or not. From the “D2 dataset”, 400 images were randomly selected for the expert opinion dataset (D3) and shown to four experienced banana harvesters. Each expert was asked to label 100 images through a custom-built Streamlit interface by choosing one of three decisions: “Cut now,” “Keep,” and “Wait more than three weeks.” Their responses were automatically saved into the main folder “Dataset (D3)” and, inside that folder, each expert has their own folder (e.g., Cutter_1, Cutter_2, Cutter_3 and Cutter_4), and inside, each of these, were three subfolders: Cut, Keep, and Wait > 3 weeks. This structure allows researchers to explore both consistency and variation in human decision-making. All three datasets were curated and cleaned to ensure label consistency and provide high-quality data for training and evaluating machine learning models.

## Limitations

This dataset has certain limitations. Images showing banana bunches covered with plastic or heavily hidden by leaves were excluded to simulate the view typically available to harvesters after clearing obstructions. As a result, the dataset may not perform well in situations with substantial occlusion. Labels were provided by expert annotators, but subjective interpretation of visual cues may lead to variation in labeling. Additionally, data collection was limited to Madeira Island, Portugal. While seasonal variation is included, environmental and agricultural conditions may differ significantly in other banana-growing regions. Furthermore, there are additional limitations, such as the relatively small sample size and the low number of experts involved in annotation.

Although experts labelled the images, some mistakes may still be present. This risk was reduced by removing 662 unclear images from D1, and by using four experts in D3 to show where opinions differ. The data come from Cavendish bananas grown in Madeira and were captured with four smartphone models, so results may not fully represent other varieties, regions, or cameras. Harvest timing depends on weather and season: in summer**,** higher temperatures speed maturation so bunches are typically cut earlier; in winter, cooler conditions slow maturation so bunches are more often kept longer. This seasonal variability may affect visual cues used for readiness and is retained to reflect real field conditions. Images also vary by time of day, lighting (such as sun, overcast and shade), weather, humidity, occlusion, hand-held viewing angles (1–3 m), backgrounds, and smartphone model, which may affect model generalization. In D2**,** the classes are not perfectly balanced (Cut 1143, Keep 1542), and the number of experts is limited, which can influence training and evaluation. D3 uses image-only ratings from four experts under a standard scenario. Because there are no touch or field cues, agreement can be lower than in the field. D3 is a 400-image subset of D2, so agreement estimates are less precise. Per-expert labels are provided so users can compute agreement or make consensus and soft labels if needed. For two-class comparisons, the three D3 options can be mapped to D2 (Cut now to Cut; Keep / Wait >3 weeks to Keep), which is simpler but loses some detail. D2 predicts Cut or Keep from D1 crops with number plates removed. Any tiny grey piece at the edge is field hardware (clip or support pin), not a label cue, and appears in both classes; this can be mitigated by masking a thin border or using occlusion-style augmentations. Users are advised to consider class-aware training, test on data from other regions, devices and varieties, interpret results with awareness of label uncertainty.

## Dataset Availability

This dataset is publicly available under Open Data Commons Attribution License v1.0 via a Mendeley data repository [20,23] and Creative Commons Attribution 4.0 International via a Zenodo [24].

## Ethics Statement

The authors have read and follow the ethical requirements for publication in Data in Brief. The authors confirm that the present work does not involve human participants, animal experimentation, or data collected from social media platforms.

## Credit Author Statement

**P. Baglat:** Data curation, Writing, Original draft preparation, Conceptualization, Methodology, Investigation, **F. Mendonça, S.S. Mostafa, F. Morgado-Dias:** Data curation, Visualization, Reviewing and Editing, Supervision.

## Data Availability

ZenodoBanana Bunch Dataset: Multi-Field Acquisition with Various Environmental Conditions (Original data)
Mendeley DataBanana Bunch Harvesting Dataset (Original data)
Mendeley DataBanana Bunch Harvesting Expert Dataset (Original data)
